# Buyang huanwu decoction combined with probiotics or prebiotics for functional recovery from stroke: A meta-analysis protocol for systematic review

**DOI:** 10.1097/MD.0000000000028371

**Published:** 2021-12-23

**Authors:** Runyu Liang, Qiang Tang, Linjing Wang, Peng Yue, Luwen Zhu

**Affiliations:** a Heilongjiang University of Chinese Medicine, Harbin, Heilongjiang, China; b Second Affiliated Hospital of Heilongjiang University of Chinese Medicine, Harbin, Heilongjiang, China; c Fourth Affiliated Hospital of Heilongjiang University of Chinese Medicine, Harbin, Heilongjiang, China.

**Keywords:** buyang-huanwu decoction, gut-brain axis, meta-analysis, stroke, traditional Chinese medicine

## Abstract

**Background:**

Stroke is a global disease that compromises human health. Considering the side effects of Western medicine, alternative medicine, such as Chinese medicine, is widely used. Concurrently, the research and development on the microbiota-gut-brain axis in recent years have made intestinal microflora the new target of treatment. We aim to scientifically evaluate the advantages and clinical guidance of using Buyang-Huanwu (BYHW) decoction combined with probiotics in the intestinal microflora.

**Methods:**

The search will focus on published Randomized Controlled Trial (RCTs) that used BYHW decoction, probiotics, prebiotics, synbiotics, or similar microecological preparations to treat stroke. We will search for relevant studies in six databases: PubMed, Embase, Cochrane Library, Web of Science, China National Knowledge Infrastructure, Wanfang Data, and Chongqing VIP Information. The retrieval date will be limited to the period from inception to June 2021 and will not be restricted by language. The extracted data will be subjected to systematic review and meta-analysis to evaluate its clinical advantages and efficacy. Patient-centred and most responsive outcomes will be selected as major outcomes, including the Fugl-Meyer (FMA) and Barthel scales. Secondary outcomes will be clinically assessed factors, including inflammatory factors in serum, platelet aggregation, other laboratory parameters, and the number and distribution of flora in the gut. We will evaluate the bias of each included study using the latest version of the Cochrane Handbook and the RoB tool. The analysis of all data and the drawing of forest maps will be performed using STAT 15.1 SE software. Regardless of the *I*^2^ values generated between the studies, we will perform a subgroup analysis. The grouping method will be based on all included research characteristics and factors that may cause heterogeneity, and may depend on differences in intervention methods, sources of subjects, and other relevant factors.

**Results:**

We plan to present the results of this systematic review in a peer-reviewed scientific journal, conferences, and popular press.

**Conclusion:**

The efficacy and safety of Buyang-Huanwu decoction combined with probiotics for the treatment of stroke will be evaluated, and the conclusion will be published to provide medical evidence for a better clinical decision of patients with stroke.

## 1 Introduction

Stroke is endangering human health because of its high fatality and disability rates,^[1]^ and its incidence is increasing.^[2]^ Further, stroke can produce limb, neurological, and autonomic dysfunction, and other sequelae, seriously affecting the quality of life.^[3]^ With a deep understanding, the prevention of stroke and the treatment of its sequelae have gradually produced a new treatment strategies.^[4,5]^ However, despite this, the treatment of stroke remains a challenging issue. The role of Western medicine is irreplaceable in the treatment of its acute stage, but the use of anticoagulants that increase the risk of bleeding cannot be ignored. Therefore, traditional Chinese medicine is widely used,^[6]^ because of its minimal side effects, and its achievement of good curative effects in clinical use.^[7]^ The Buyang-Huanwu (BYHW) decoction, for example, is the most commonly used Chinese herbal medicine for stroke,^[8]^ and has a satisfactory effect on the treatment of stroke sequelae.^[9]^

In recent years, the microbiota-gut-brain axis has been found to be closely related to the nervous and gastrointestinal systems,^[10]^ and has gradually become a topic of interest in research. In addition, the microbiota-gut-brain axis is a 2-way regulating system between the gut and the brain. Improving the gut flora can effectively reduce inflammation and improve immune response after stroke,^[11]^ indicating that it is expected to be a new strategy in treating stroke sequelae.^[12]^ It has been pointed out that intestinal dysfunction in stroke patients is a poor prognostic factor, and a reasonable regulation of intestinal flora effectively reduces gastrointestinal dysfunction and risk factors.^[13]^ Probiotics have been reported to improve the microbial profile of the gut and have shown good results in some randomized controlled trials (RCTs).^[14]^

Although BYHW decoction^[15]^ and probiotics^[16]^ can yield good clinical results individually, there is no evidence on the effect of the combined use of BYHW decoction and probiotics. This study aims to prove the effectiveness and advantages of the combined use of BYHW decoction and probiotics through a meta-analysis.

## 2 Methods and analysis

### 2.1 Design

The search will focus on published RCTs that used BYHW decoction, probiotics, prebiotics, synbiotics, or similar microecological preparations to treat stroke. The extracted data will be subjected to a systematic review and meta-analysis to evaluate its clinical advantages and efficacy. The research program will be registered in PROSPERO(CRD42021257990).

### 2.2 Eligibility criteria

#### 2.2.1 Inclusion criteria

RCTs using the BYHW decoction in combination with or without probiotics or prebiotics to treat stroke will be included. Patients included in the trial will be determined by relevant guidelines and supported by imaging, but the age, duration, and location of the lesion will not be limited. The patients receiving thrombolysis and other treatments will be included, but at least 1 main outcome must be included in the study outcome. In addition, conventional treatment and nursing methods will not be restricted.

#### 2.2.2 Exclusion criteria

Patients who have been prescribed or are being treated with probiotics and prebiotic drugs before being included in the trial, or have major diseases, such as tumors, myocardial infarction, and other major diseases, or those who have consciousness disorders, will be excluded. Trials with unclear intervention methods that do not match the main results will be excluded. In addition, duplicate studies will also be excluded.

### 2.3 Types of outcome measures

#### 2.3.1 Primary outcome

Patient-centered and most responsive outcomes will be selected as major outcomes, including the Fugl-Meyer (FMA) and Barthel scales.

The FMA scale is widely used to assess limb function in stroke patients.^[17,18]^ The independent evaluation of upper and lower limb items on the scale can clearly reflect the patient's limb movement performance and movement quality.

Barthel scale is a reliable measure of a patient's ability to live daily life and can also explain changes in physical function.^[19]^ The ability of daily living of patients has been evaluated in many ways through the evaluation of the function of eating, dressing, and walking.

#### 2.3.2 Secondary outcomes

Secondary outcomes will be clinically assessed factors, including inflammatory factors in serum, platelet aggregation, and other laboratory parameters. In addition, considering the use of probiotics, the number and distribution of flora in the gut will be considered as outcomes of treatment. Different trials often use different clinical or laboratory test data as outcome indicators. Here, we recommend the selection of indicators that can reflect changes in the disease to some extent or that are easily explained to people.

### 2.4 Search methods for study identification

**Figure 1:**
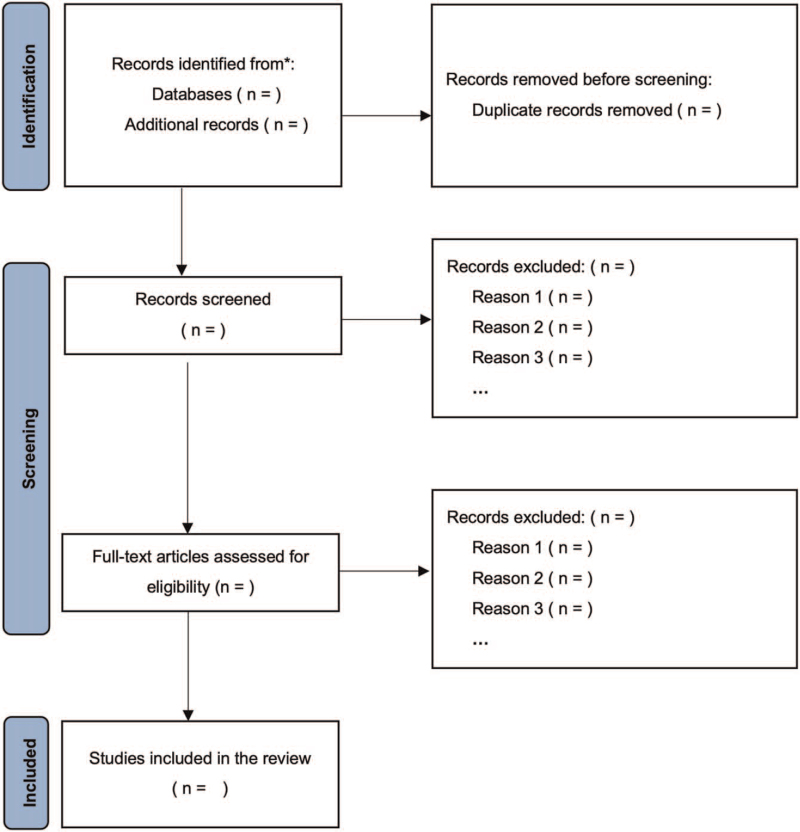
Preferred reporting items for systematic review and meta-analysis flow.

We will search for relevant studies in 6 databases: PubMed, Embase, Cochrane Library, Web of Science, China National Knowledge Infrastructure, Wanfang Data, and Chongqing VIP Information. The retrieval date will be limited to the period from inception to June 2021 and will not be restricted by language. Keywords will include stroke, cerebral ischemia, cerebral vascular accident, Buyang Huanwu decoction, probiotics, and prebiotics. Further, in order to ensure the comprehensiveness of the data, we will also search for unpublished trials or data that exist in conferences or in ClinicalTrials.gov. The specific retrieval methods and flowcharts^[20]^ are shown in Table 1 and Figure 1.

**Table 1:** Search strategy in English databases (using PubMed as an example).

Search	Search Strategy
#1	“Stroke” [MeSH]
#2	“Stroke” OR “Brain Infarction” OR “Cerebrovascular Accident” OR “Brain Ischemia”
#3	#1 OR #2
#4	“Probiotics”[Mesh] OR “Prebiotics”[Mesh] OR “Synbiotics”[Mesh]
#5	“Probiotics” OR “Prebiotics” OR “Synbiotics” OR “Probiotic∗” OR “Prebiotic∗” OR “Synbiotic∗”
#6	#4 OR #5
#7	“Bu Yang Huan Wu” OR “Bu Yang Huan Wu decoction” OR “BYHW” OR “BYHW decoction”
#8	#6 OR #7
#9	#3 AND #8

MeSH = Medical Subject Headings.

### 2.5 Data collection and analysis

#### 2.5.1 Extraction and management of data

According to the scheme designed in advance, 2 reviewers will first reprocess the retrieved studies using Endnote X9 (Clarivate Analytics, PA), then screen and evaluate the remaining studies, and check the results against each other. If different opinions arise, a third evaluator will be introduced to judge the results. We will attempt to address missing data or wrong data using a variety of methods. We will prioritize contacting the author. In cases where contact cannot be established, if available, we will preferentially use data from other studies that can be used for statistics. If the result data do not contain standard deviation (SD), it will be calculated according to the method of Furukawa et al.^[21]^ If none of the above methods resolve the problem, the study may be excluded.

We will extract data on publication dates, trial locations, sample information, intervention modalities, treatment outcomes, and conclusions from eligible studies.

#### 2.5.2 Quality assessment of the included literature

Although an RCT is considered to have a high degree of credibility of evidence, the implementation and reporting of bias cannot be avoided, and it is necessary to assess the quality. We will evaluate the bias of each included study using the latest version of the Cochrane Handbook^[22]^; the use of the RoB^[23]^ tool will also be necessary. The outcome of the evaluation tool will be categorized as low, unclear, or high bias. If possible, we will eliminate the studies with high bias to ensure that the results are true and credible. The same steps will be adopted by both researchers.

### 2.6 Data analysis

The analysis of all data and the drawing of forest maps will be performed using STAT 15.1 SE software (Stata Corp, TX). For binary variables, the rate ratio/risk ratio (RR) is recommended for effect scale, because RR can be better described and explained. For continuous variables, the weighted mean difference (WMD) will be selected, and it is more appropriate to use WMD for the results measured by the same method. Regardless of whether it is a binary or continuous variable, the random effects model is preferred, which reduces the variability of internal research. If there are fewer studies (n < 5), it is more reasonable to choose a fixed-effects model because the variance between studies is not easy to estimate accurately.

### 2.7 Heterogeneity assessment

Variability between studies in meta-analysis is called heterogeneity and can be caused by a variety of factors. The new edition of the Cochrane Handbook^[22]^ does not recommend the selection of an effect model based on heterogeneity; therefore, we will consider only the heterogeneity shown under the random effects model. *I*^2^ > 25 is generally considered heterogeneous, and *I*^2^ > 75 is considered to be highly heterogeneous. When heterogeneity occurs, it will be necessary to analyze the source of heterogeneity and choose appropriate methods to eliminate it. The origin of heterogeneity will be analyzed based on the methodological and clinical heterogeneity. Methodological heterogeneity may arise from differences in study design and measurement methods, and differences between study subjects and interventions will be considered clinical heterogeneity.

### 2.8 Subgroup analysis

Regardless of the *I*^2^ values generated between the studies, we will perform a subgroup analysis. The grouping method will be based on all included research characteristics and factors that may cause heterogeneity, and may depend on differences in intervention methods, sources of subjects, and other relevant factors. For the results for which the sources of heterogeneity cannot be clarified after subgroup analysis, only a systematic review will be used to describe the results of the study.

In addition, if the included study contains sufficient data related to the intestinal flora to be analyzed, a subgroup analysis will be performed according to the different prebiotics used.

### 2.9 Sensitivity analysis

A sensitivity analysis will be used to verify the stability of the results of this study. STAT 15.1 will be used to calculate the effect size when any of the studies included in the analysis are excluded. If the 95% confidence interval (95% CI) and effect size results obtained have not changed, it indicates that the results are robust, and if they have changed, they indicate high sensitivity and unstable results, and the source of disputes needs to be further clarified.

### 2.10 Assessment of publication bias

We will analyze and study the bias situation and use the Begg's method for quantitative testing. If the number of studies included is small (n < 8), the Egger's test method is more accurate. If the results are obviously biased, the stability of the results will be verified by the metatrim.

### 2.11 Strength of evidence

Quality evaluation of all outcome indicators will be based on the Grading of Recommendations Assessment, Development and Evaluation tool (GRADE),^[24]^ and the results will include high, moderate, low, and very low evidences. As high-level evidence, the initial level of RCT will be 4 (high), and every downgrading factor will reduce the level of evidence by 1 level. Degradation factors include limitations, inconsistencies, indirectness, publication bias, and imprecision. Low evidence strength can make the results unreliable (Table 2).

**Table 2:**
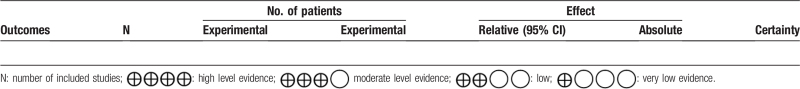
The GRADE summary.

## 3 Discussion

Researchers have been actively seeking new methods to treat stroke and its sequelae. The use of early thrombolysis and anticoagulant and antiplatelet drugs has greatly increased the survival rate and improved its prognosis. Although these drugs play a positive role, they increase the risk of bleeding in patients.^[25,26]^ Patients question the side effects of certain drugs and do not always adhere to the medication. Reasonable adjustments and optimization of treatment plans in patients are also key to treatment. In recent years, the treatment of stroke has gradually diversified. In particular, alternative medicine, such as sports, rehabilitation, traditional Chinese medicine, acupuncture, and moxibustion have been widely used and accepted in clinical treatment because of their less side effects and convenience customizing according to the patient.^[4]^ Unlike the use of antiplatelet drugs, such as clopidogrel and aspirin, the early and reasonable use of alternative medicine, such as traditional Chinese medicine and acupuncture, does not increase the risk of bleeding.^[27,28]^ Similarly, prebiotics, probiotics, and synbiotics^[14]^ have gradually become the new drugs for the prevention and treatment of stroke through the microbiota-gut-brain axis, and have shown potential in research.^[29,30]^

The recovery of stroke is a multicellular process, including, but not limited to, neuronal cells involved and cells involved in the inflammatory response or a large number of immune cells. Improvement of intestinal flora can promote recovery after stroke by regulating the immune response.^[31]^ In addition, BYHW decoction and some traditional Chinese medicines commonly used in stroke treatment have been reported to promote angiogenesis,^[32,33]^ and can also regulate cell autophagy to reduce damage.^[34]^

Although some basic studies have confirmed the benefits of BYHW decoction and probiotics in promoting the recovery of stroke, there is still a lack of clinical trials to verify the advantages of their combined use. This study will extract and analyze data in strict accordance with the requirements of relevant guidelines, aims to guide its clinical application through meta-analysis, and provide reliable results to evaluate the validity and strength of evidence.

### 3.1 Strength and limitations of this study

The meta-analysis will evaluate the efficacy of BYHW combined with prebiotics, probiotics and other microbial preparations on stroke sequelae.

The plan explains the selection method of the results. On the one hand, it selects the index that best reflects the changes in the patient's condition, and on the other hand, considers the changes in the intestinal flora of the patient after taking microbial preparations from the intestinal flora.

At the same time, when choosing the effect scale, the protocol also made suggestions. This avoids incorrect use of analytical methods or problems in presenting results.

This protocol attempts to use the brain-gut axis as a treatment strategy to explain BYHW combined with prebiotics as a basis for clinical treatment.

When selecting the database, only researches published in English and Chinese are searched, which may cause language bias.

## 4 Ethics and dissemination

The meta-analysis is based on all published studies and does not require patients to participate and use their data, so there is no need to worry about patient privacy and ethics committee review. We plan to present the results of this systematic evaluation in peer-reviewed scientific journals, conferences, and the mass media.

## Acknowledgments

The authors are grateful to PhD Qiang Tang and PhD Luwen Zhu for their helpful assistance.

## Author contributions

Both Runyu Liang and Qiang Tang are the first authors. Runyu Liang and Qiang Tang designed the protocol, Runyu Liang wrote the manuscript, Linjing Wang and Peng Yue participated in material collection and Statistical analysis of data, Luwen Zhu designed experimental methods and revised the manuscript. All authors read and approved the final manuscript.

**Conceptualization:** Luwen Zhu, Peng Yue.

**Methodology:** Runyu Liang, Linjing Wang.

**Resources:** Linjing Wang.

**Writing – original draft:** Runyu Liang, Qiang Tang.

**Writing – review & editing:** Qiang Tang, Luwen Zhu.
